# Aeroportia and pneumatosis intestinalis in infants with food protein‐induced‐allergic proctocolitis: A presentation of two cases

**DOI:** 10.1002/jpr3.12164

**Published:** 2025-01-03

**Authors:** Jessica Ruesen, Clarissa Gaitan Villela, Theodoros Xydias, Freya Kramer, Corinne Daester, Henrik Koehler

**Affiliations:** ^1^ Department of Pediatrics KSA Children's Hospital Aarau Aarau Switzerland; ^2^ Department of Pediatric Radiology Cantonal Hospital Aarau Aarau Switzerland; ^3^ Department of Neonatology KSA Children's Hospital Aarau Aarau Switzerland; ^4^ Medical Faculty University of Basel Basel Switzerland

**Keywords:** aeroportia, food protein‐induced‐allergic proctocolitis, non‐IgE‐mediated gastrointestinal food allergies, pneumatosis intestinalis

## Abstract

Bloody stools are a particularly concerning symptom in neonates and infants. The differential diagnosis reaches from life‐threatening to benign conditions. We would like to present two infants, who presented to the paediatric emergency department with bloody stools and showed pneumatosis on ultrasonography, which initially led to the suspicion of a potentially life‐threatening condition. Both children had an unremarkable physical examination and both the laboratory chemical parameters, as well as the stool analyses were without abnormal findings. As in summary, there was no evidence of necrotizing enterocolitis, and Food Protein‐Induced Allergic Proctocolitis (FPIAP) was considered the most likely diagnosis, an ambulant nutritional counselling was provided. As FPIAP is a clinical diagnosis, the number of cases in which pneumatosis can be detected is unclear. It is speculated that, like atopic dermatitis, food allergies alter, for example, the tight junctions and lead to an increased intestinal permeability which might result in pneumatosis intestinalis.

## 1 INTRODUCTION

The differential diagnosis from bloody stool in infants and neonates reaches from life‐threatening to benign conditions (Table 1).

**Table 1:** Differential diagnoses of bloody stools in infants.

Anal fissure
Coagulopathy
Food protein‐induced allergic proctocolitis
Infectious colitis
Inflammatory bowel disease
Intussusception
Meckel's diverticulum
Necrotizing enterocolitis

Food protein‐induced allergic proctocolitis (FPIAP) being one of the more common causes in outpatients is often encountered by paediatricians.^1^ Its prevalence varies greatly depending on the study and region. Bloody stools present usually within the first 2 months of life without other symptoms in children in good general condition.^2^ The most common trigger is cow's milk protein.^3^ Due to its generally benign outcome, FPIAP is considered a clinical diagnosis.

The simplest diagnostic tool and treatment in breast‐fed infants is excluding cow's milk protein in the maternal diet. This requires good education and nutritional counselling, because of possible cross reactions.^3^ Improvement of symptoms might take some weeks.

Pneumatosis intestinalis (PI), pneumo‐/aerobilia and aeroportia are radiologic findings that reflect the presence of air in the intestinal wall, bile ducts or portal vein. These can be seen in a well‐performed sonography and are rarely found in children. In neonates or infants, the primary differential diagnosis is necrotizing enterocolitis (NEC), a life‐threatening condition.^4^

We describe two cases of patients with FPIAP presenting with aeroportia and pneumatosis intestinalis. In both cases, an ultrasound was conducted to exclude a serious cause of hematochezia, for example, intussusception.

## 2 CASE PRESENTATIONS

### 2.1 Case A

A 25‐day‐old male neonate, born by caesarean delivery because of maternal exhaustion at 41 2/7 weeks' gestation with a birthweight of 3910 g was presented to the emergency department on two consecutive days with first‐time bloody stools. The patient had received antibiotics (amoxicillin and gentamycin) in hospital at the age of 20 to 21 days due to a febrile infection. He was fully breastfed. The clinical examination was inconspicuous in every aspect. The blood work showed normal values.

**Figure 1:**
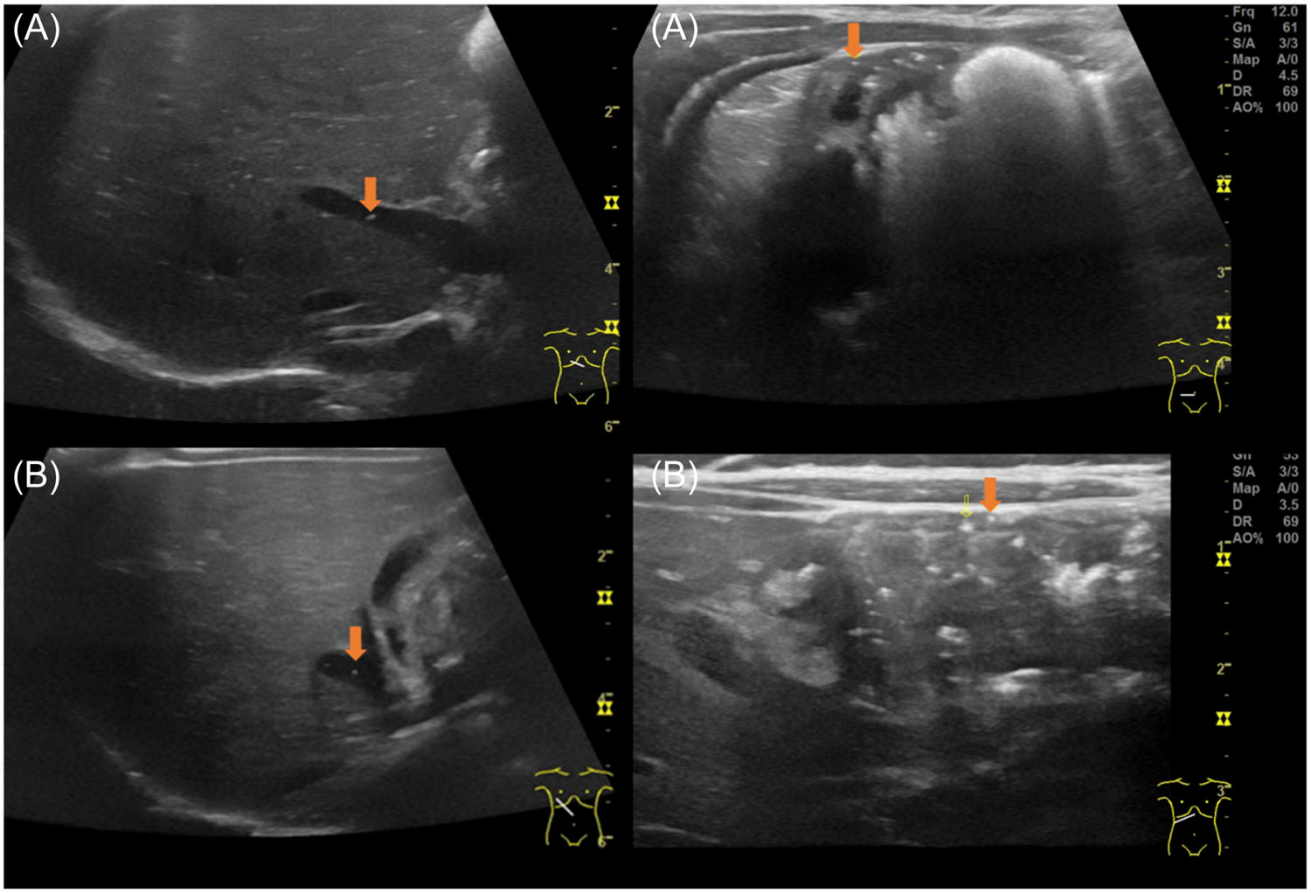
Ultrasound findings of aeroportia (left side) and pneumatosis intestinalis (right side) in patient A and patient B.

An ultrasound showed pronounced aeroportia and minor PI (Figure 1). The patient was hospitalised for observation and no other therapeutic measures were taken apart from the implementation of a diet free of cow's milk protein. His clinical condition remained unchanged with unremarkable laboratory tests and negative stool tests for pathogens. Overall, there was no evidence of NEC, and FPIAP was the suspected diagnosis. Before discharge, nutritional counselling was provided to the mother regarding a diet free of cow's milk and soy protein.

In an outpatient follow‐up after 10 days, the patient was in good health, his stools contained only minor traces of blood, and an ultrasound showed only residual aeroportia and PI.

### 2.2 Case B

A 4‐month‐and‐6 day‐old male infant was presented to the emergency department with bloody stools for the past 26 days and an episode of pronounced crying on the day of presentation. He was born by caesarean section due to suspected Triple I (intrauterine inflammation, infection or both) at 39 2/7 weeks' gestation with a birthweight of 3440 g.

He was fully breastfed until the approximate age of 2 months. On trying different formula milk and goat's milk with the intention of weaning, bloody stools and occasional vomiting occurred a few days after milk exposure. Otherwise, he showed an appropriate progress with good weight gain. The mother omitted cow's milk for 2 weeks but not soy products.

The patient was in good clinical condition and the clinical examination showed no anomalies apart from a slightly distended abdomen.

The blood work and stool examination for pathogens were inconspicuous (Table 2). An ultrasound revealed PI in the transverse colon and aeroportia, without any other conspicuities (Figure 1).

**Table 2:** Laboratory values of presented cases.

	Patient A	Patient B
Haemoglobin	145 g/L	111 g/L
Platelets	131 × 10^9^/L	628 × 10^9^/L
Leukocytes	8.3 × 10^9^/L	16 × 10^9^/L
Eosinophiles	‐	2.45 G/L
INR	1.0	‐
CRP	Not elevated	Not elevated
PCR Stool Panel	Negative	Negative

Abbreviations: CRP, C‐reactive protein; INR, international normalized ratio; PCR, polymerase chain reaction.

The parents were informed about FPIAP, nutritional counselling was organised, and a dietary restriction of milk and soy was recommended.

The follow‐up 2 months later showed an infant with unremarkable stools and a normal abdominal ultrasound.

## 3 DISCUSSION

These two cases of infants with FPIAP show that alarming signs on imaging must always be taken in the clinical context. In contrast to the potential life‐threatening diseases, especially NEC, infants with FPIAP present in good clinical condition with inconspicuous laboratory results.

The prevalence of PI and/or aeroportia in infants with FPIAP is unknown, as most children with suspicion of FPIAP do not undergo any diagnostic imaging.

In contrast to FPIAP, other non‐IgE‐mediated gastrointestinal food allergies (non‐IgE‐GI‐FAs), namely Food Protein Induced Enterocolitis Syndrome (FPIES) and Food Protein‐induced enteropathy, affect the whole and the upper gastrointestinal tract, and the infants often present in reduced general condition and with failure to thrive.^5^

PI and aeroportia have been observed in infants with cow's milk protein allergy and, especially in infants with FPIES, have led to the misdiagnosis and primary treatment as a NEC, with fasting periods and intravenous antibiotics.^6^ The underlying pathophysiology leading to these ultrasound findings in non‐IgE‐GI‐FAs is not understood, as the exact pathophysiology for non‐IgE‐GI‐FAs is not known. It remains unclear why our patients developed PI and aeroportia as FPIAP by definition specifically affects the colon. Given their symptoms and clinical course we considered FPIAP for these findings.

In histopathological samples from children with non‐IgE‐GI‐FAs more eosinophils are present in the affected parts of the intestine. Altered cytokine levels and certain T‐cell reactions seem to play a role in the pathogenesis of non‐IgE‐GI‐FAs. Studies have shown an increased prevalence of atopic dermatitis in children with non‐IgE‐GI‐FAs.^7^ In non‐IgE‐GI‐FAs and in atopic dermatitis the release of cytokines causes a barrier defect. In atopic dermatitis this causes a lack of filaggrin. There is no filaggrin in the intestinal mucosa, but studies described a change in the tight junctions due to higher TNF‐α concentration present in patients with food allergies.^1^ Transforming growth factor‐β (TGF‐β) has also been shown to be important for the intestinal barrier function: a decrease of TGF‐β could be involved in the pathogenesis of non‐IgE‐GI‐FAs. These immunological changes could explain an increased intestinal permeability and could confirm the mechanical theory of the pathogenesis of PI.^8^ This mucosal damage is also thought to be an underlying mechanism leading to aeroportia.^9^ The bacterial theory of gas‐forming bacteria entering through fissures and the submucosa is less likely, as the few histopathological samples from patients with these allergies showed no evidence of bacteria in the submucosa.^10^

The therapeutical approach in FPIAP consists of an elimination diet for the mother in children receiving human milk or hypoallergenic formula for formula‐fed children. However, in mild cases, a watch‐and‐wait approach gains more popularity due to the self‐limiting nature of the disease.^5^ In the presented cases, an improvement of PI and aeroportia was observed as well after implementation of the appropriate diet.

In conclusion, PI and aeroportia are associated with potential life‐threatening diseases, especially in neonates, where the primary diagnosis associated with these radiological findings is NEC. Our cases illustrate that these findings can also be present in neonates or infants with benign conditions like FPIAP presenting with a good clinical course without systemic deterioration and improvement after implementation of the elimination diet. The treatment of infants presenting with PI and aeroportia should vary depending on the clinical presentation. The implementation of a diet without further therapeutical measures should only be considered in infants presenting in good clinical condition and without other risk factors for NEC. To confirm a causal relationship between FPIAP and PI and aeroportia further research is needed.

## CONFLICT OF INTEREST STATEMENT

The authors declare no conflict of interest.

## ETHICS STATEMENT

Written informed consent was obtained by the parents of the patients for publication of the case details.

## References

[1] Labrosse R , Graham F , Caubet J‐C . Non‐IgE‐mediated gastrointestinal food allergies in children: an update. Nutrients. 2020;12:2086. 32674427 10.3390/nu12072086 PMC7400851

[2] Martin VM , Virkud YV , Seay H , et al. Prospective assessment of pediatrician‐diagnosed food protein‐induced allergic proctocolitis by gross or occult blood. J Allergy Clin Immunol Pract. 2020;8:1692‐1699. 31917366 10.1016/j.jaip.2019.12.029 PMC8403015

[3] Meyer R , Venter C , Bognanni A , et al. World Allergy Organization (WAO) Diagnosis and Rationale for Action against Cow's Milk Allergy (DRACMA) guideline update—VII—milk elimination and reintroduction in the diagnostic process of cow's milk allergy. World Allergy Organ J. 2023;16:100785. 37546235 10.1016/j.waojou.2023.100785 PMC10401347

[4] Esposito F , Mamone R , Di Serafino M , et al. Diagnostic imaging features of necrotizing enterocolitis: a narrative review. Quant Imaging Med Surg. 2017;7:336‐344. 28812000 10.21037/qims.2017.03.01 PMC5537125

[5] Zhang S , Sicherer S , Berin MC , Agyemang A . Pathophysiology of non‐IgE‐mediated food allergy. Immunotargets Ther. 2021;10:431‐446. 35004389 10.2147/ITT.S284821 PMC8721028

[6] Hernández‐Almeida P , Vásconez‐Muñoz F , Vásconez‐Montalvo A , Sempértegui‐Moscoso R , Contreras G , Carrión‐Jaramillo E . Food protein‐induced enterocolitis syndrome with pneumatosis intestinalis in an exclusively breastfed infant: a case report and literature review. Clin Case Rep. 2022;10:e6520. 36439383 10.1002/ccr3.6520 PMC9684614

[7] Caubet J‐C , Nowak‐Węgrzyn A . Current understanding of the immune mechanisms of food protein‐induced enterocolitis syndrome. Expert Rev Clin Immunol. 2011;7:317‐327. 21595598 10.1586/eci.11.13

[8] Pieterse AS , Leong ASY , Rowland R . The mucosal changes and pathogenesis of pneumatosis cystoides intestinalis. Hum Pathol. 1985;16:683‐688. 4007844 10.1016/s0046-8177(85)80152-0

[9] Iskander OA . Unraveling the mystery of hepatic portal vein gas: exploring its benign nature and surgical implications. Cureus. 2023;15:e41231. 37529512 10.7759/cureus.41231 PMC10387453

[10] Abboud B , Hachem JE , Yazbeck T , Doumit C . Hepatic portal venous gas: physiopathology, etiology, prognosis and treatment. World J Gastroenterol. 2009;15:3585‐3590. 19653334 10.3748/wjg.15.3585 PMC2721230

